# Clinical remission of feline sino-nasal aspergillosis despite evidence of persistent infection

**DOI:** 10.1177/20551169231201605

**Published:** 2023-10-03

**Authors:** Jack Fawsitt, Oliver Russell, Akash Alexander, Anne-Lorraine Peschard, Hannah Wong, Andre Kortum

**Affiliations:** The Queen’s Veterinary School Hospital Cambridge University Veterinary School, Cambridge, UK

**Keywords:** Sino-nasal aspergillosis, clinical remission, multimodal therapy, *Aspergillus versicolor*, *Aspergillus fumigatus*

## Abstract

**Case summary:**

Feline sino-nasal aspergillosis is a rare condition with only sparse heterogeneous reports in the literature regarding its treatment. This report describes the presentation, treatment and outcome of a cat with sino-nasal aspergillosis treated by meticulous debridement in combination with topical and systemic azole therapy. Diagnosis was based on MRI, in combination with rhinoscopic assessment and visualisation of fungal plaques, followed by histopathology, fungal culture and panfungal PCR. The cat was treated by debridement of fungal plaques via anterior rhinoscopy and frontal sinusotomy and local instillation of 1% clotrimazole solution, followed by a 4-week course of oral itraconazole. Histopathology confirmed fungal rhinitis and culture identified *Aspergillus fumigatus* and *Aspergillus versicolor*. Clinical remission was achieved after treatment; however, evidence of persistent infection was confirmed in the post-mortem examination 8 months after the cat was euthanased for unrelated reasons.

**Relevance and novel information:**

Despite clinical remission, the persistence of fungal infection post mortem highlights the challenges of monitoring the response to treatment and illustrates that the resolution of clinical signs does not necessarily equate with a disease cure.

## Introduction

Sino-nasal aspergillosis (SNA) is a rare condition in cats, typically characterised by sneezing, nasal discharge and nasal turbinate destruction.^1^ It is distinguished from the more invasive sino-orbital aspergillosis (SOA) by the lack of orbital and surrounding tissue invasion; however, it has been suggested that SOA may represent an extension of SNA caused by the more invasive *Aspergillus* species.^2,3^ Owing to its rarity, the evidence base regarding feline SNA is limited compared with the canine and human literature.^4^ Furthermore, previous guidelines on the treatment of feline SNA are often conflicting, some stating that systemic antifungal agents are required in combination with local therapy,^5^ and others stating that systemic therapy is only required if there is histological evidence of mucosal invasion by fungal hyphae or culture of *Aspergillus felis*.^4,6,7^

This case report describes the treatment and outcome of a rare condition of feline SNA resulting from *Aspergillus versicolor* and *Aspergillus fumigatus* co-infection. Treatment involved debridement in combination with topical and systemic azole therapy with repeat MRI. Since this is a rare condition with no previous reports of feline SNA from *A versicolor*, this case report aims to contribute clinically relevant data to the current limited literature.

## Case description

**Figure 1:**
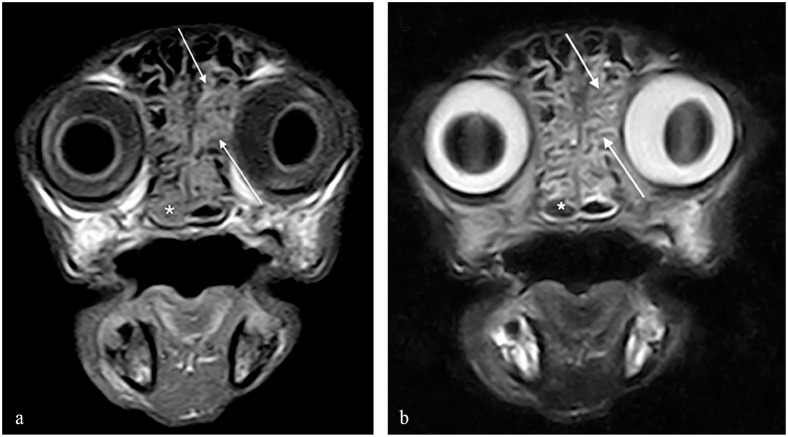
Transverse MRI scans at the level of the caudal nasal turbinates. (a) A T1-weighted image is provided as well as (b) a T2-weighted image. Material is visible between the turbinates (arrows) and in the right choana (asterisk) in the normally gas-filled (signal void) space

A male neutered domestic shorthair cat aged 13 years and 10 months was presented with a 5-day history of intermittent epistaxis. Lateralisation of epistaxis could not be determined owing to paroxysms of sneezing. The patient had been investigated 19 months previously for sneez-ing, bilateral purulent nasal discharge and intermittent epistaxis. Investigations, including MRI (Figure 1), rhinoscopy and nasal histopathology, had been consistent with chronic lymphoplasmacytic and neutrophilic rhinitis. Ulceration of the nasal planum, gingivostomatitis and glossitis had been documented and a positive feline calicivirus result obtained on PCR. After a full-mouth dental extraction, the oral lesions resolved and nasal signs were reported to have improved. Aside from a longstanding history of concurrent diabetes mellitus (well manag-ed with lente insulin 3.0 IU SC q12h, Caninsulin; MSD Animal Health), there was no other pertinent medical history, including no travel outside of the UK.

**Figure 2:**
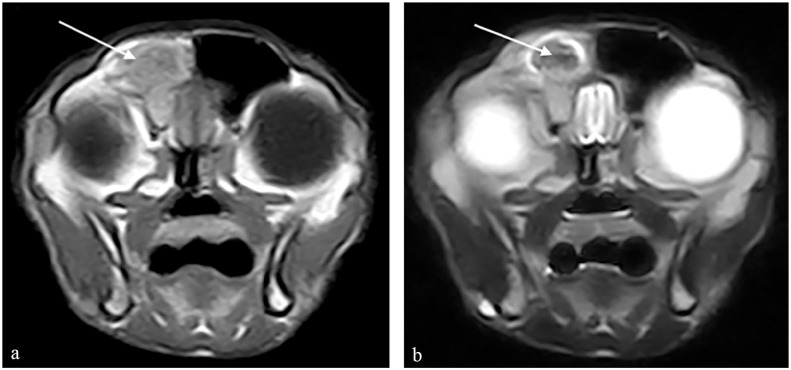
Transverse MRI scans at the level of the caudal nasal turbinates. (a) A T1-weighted (T1W) image is provided as well as (b) a T2-weighted (T2W) image. The right frontal sinus is filled with heterogeneously T2W hypointense, T1W mildly hyperintense non-contrast enhancing material (arrow).

The clinical examination was unremarkable, with no evidence of nasal discharge, deformity, altered airflow and no pain on nasal and facial palpation. Clinicopathological investigations identified no abnormalities other than a mild mature neutrophilia (19.03 ×10^9^/l, reference interval [RI] 2.5–12.5 ×10^9^/l) and moderate, regenerative anaemia (PCV 20%, RI 26–45; reticulocytes 260 ×10^9^/l, RI 0–60 ×10^9^/l). MRI of the head documented multifocal turbinate destruction with T2-weighted (T2W) hyperintense material throughout both nasal cavities, extending into the right frontal sinus and the left sphenoid sinus (Figure 2). The right frontal sinus was filled with heterogeneously T2W hypointense, T1-weighted mildly hyperintense non-contrast enhancing material. Similar material was also present in the right ventral meatus and choana.

On rhinoscopy, turbinate destruction was evident bilaterally, along with a large volume of mucopurulent discharge. In both middle meatuses, conglomerates of cream-brown caseous material resembling fungal plaques extended into the caudal ventral meatus in the right nasal cavity and occluded the choana. The plaques were debrided and retrieved via the external nares and via the choanae into the nasopharynx. Both nasal cavities were flushed copiously with 0.9% sodium chloride. Histopathology of the nasal turbinates and plaques demonstrated a marked chronic-active ulcerative neutrophilic sinusitis with abundant intraluminal periodic acid–Schiff (PAS)-positive fungal hyphae, consistent with fungal rhinosinusitis; however, the fungal culture and PCR were negative.

Given the evidence of frontal sinus involvement on imaging, a bilateral sinusotomy was subsequently performed. A large volume of material resembling fungal plaques filled the right frontal sinus with a smaller amount of white discharge in the left sinus. This material was debrided and both sinuses were lavaged with 0.9% sodium chloride. The nasopharynx and external nares were then occluded before instilling a liquid solution of 1% clotrimazole (Canesten; Bayer) into the frontal sinus via the sinusotomy site until the nasal cavity and frontal sinus were filled. After a dwell time of 15 mins, the solution was drained via the external nares, followed by routine closure of the sinusotomy sites. The patient recovered uneventfully and was discharged with a 4-week course of itraconazole (5 mg/kg q24h PO, Itrafungol; Elanco).

**Figure 3:**
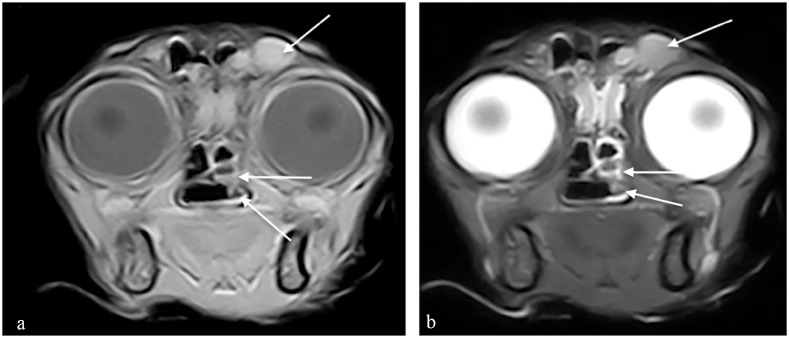
Transverse MRI scans at the level of the caudal nasal turbinates. (a) A T1-weighted (T1W) image is provided as well as (b) a T2-weighted (T2W) image. T2W and T1W hyperintense material fills the left frontal sinus (top arrow), left sphenoidal sinus (middle arrow) and the ventral left aspect of the nasopharynx (bottom arrow).

**Figure 4:**
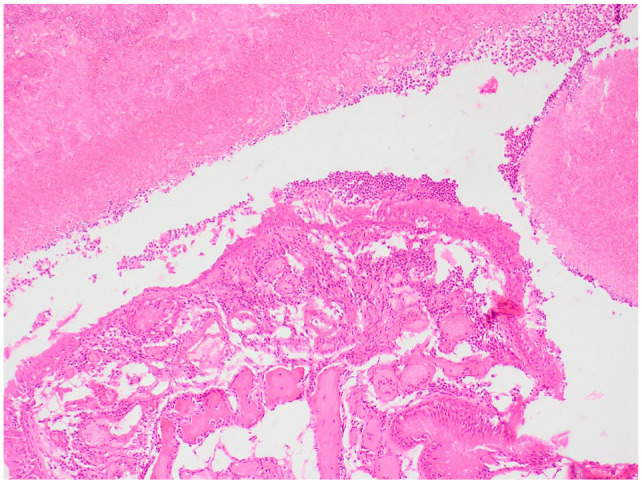
× 100 magnification haematoxylin and eosin staining of post-mortem nasal biopsy with degenerate neutrophils and proteinaceous debris

**Figure 5:**
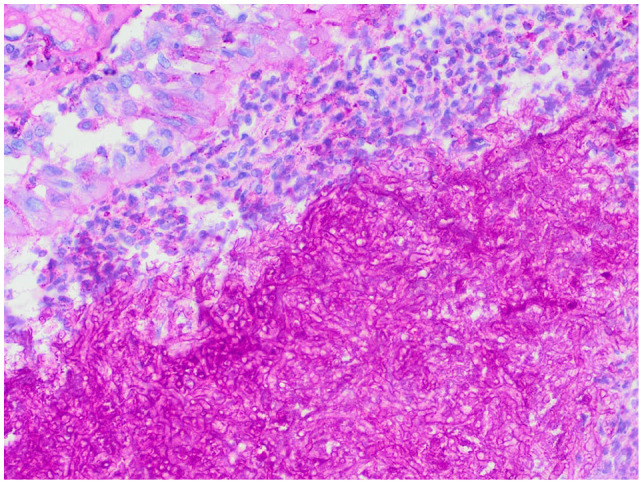
× 400 magnification of post-mortem nasal biopsy with PAS (periodic acid–Schiff) staining with PAS-positive fungal hyphae.

On reassessment after 1 month, the cat was considered to have complete resolution of clinical signs. Clinical examination was unremarkable with no evidence of nasal discharge, no disrupted nasal airflow or pain or deformity of the nasal, maxillary or frontal bones. Repeat imaging was discussed but was declined due to the cat’s clinical improvement. Itraconazole medication was discontinued, and the patient remained free of clinical signs for 8 months before re-presenting to the hospital for a 1-week history of hyporexia, lethargy, abdominal distension and a 3–4-month history of weight loss. Clinical investigations were consistent with a metastatic pancreatic adenocarcinoma and the patient was subsequently humanely euthanased. A post-mortem MRI head scan documented material within the nasal cavity, left frontal sinus, sphenoidal sinus and ventral aspect of the nasopharynx, consistent with the previous fungal rhinosinusitis (Figure 3). On necropsy, the pancreatic mass was diagnosed as a pancreatic ductal adenocarcinoma. Large fungal plaques were also identified between the left and right ethmoturbinates and both frontal sinuses were filled with firm cream tissue. The right frontal sinus contained large amounts of gelatinous fluid. Histopathology confirmed large numbers of luminal PAS-positive fungal hyphae alongside degenerate neutrophils and proteinaceous debris (Figures 4 and 5). The panfungal PCR was negative due to unidentified inhibitory factors; however, culture identified two *Aspergillus* species, *A fumigatus* and *A versicolour.*

## Discussion

Previous reports on the management of feline SNA describe the use of systemic antifungals, topical application of azole agents and debridement either in isolation or in combination;^4–6,8,9^ however, given the limited numbers of cats reported and the variability in treatment regimens, conclusions regarding treatment efficacy are difficult to draw. In this case with *A versicolor* and *A fumigatus* co-infection, the clinical presentation, imaging findings and histological changes were similar to previously reported feline SNA cases. Complete clinical remission was achieved after endoscopic and surgical debridement, topical instillation of clotrimazole and oral itraconazole therapy; however, the post-mortem findings demonstrate that clinical signs may correlate poorly with disease status and clinical remission cannot be relied upon as an indicator of disease cure.

The role of fungal plaque debridement is currently controversial. In humans, treatment of fungus balls or aspergillomas – a non-invasive fungal rhinosinusitis resembling feline and canine SNA – is managed by endoscopic or surgical debridement alone, without adjunctive topical or systemic therapy.^10–12^ Debridement is considered to be an important part of treatment in both dogs and cats; however, the evidence base to support this supposition is currently limited.^4,6,8,9,13,14^ Treatment in dogs typically involves a combination of fungal plaque debridement and topical azole therapy either by passage of nasal catheters or by frontal sinus trephination, with reported cure rates in the range of 62–94%.^13–16^ In cats, while there are reports of successful clotrimazole infusion without debridement,^17,18^ more recent literature suggests debridement is essential.^4,6^ However, in a previous case report of *A fumigatus* rhinosinusitis in a British Shorthair treated with endoscopic debridement and oral itraconazole, relapse occurred after 4.5 months, necessitating repeat debridement and further oral therapy.^8^ While the reasons for recurrence are poorly understood, this may be partly related to the smaller size of the feline skull; complete debridement is more difficult to achieve endoscopically, particularly if the disease involves the caudal nasal cavity or paranasal sinuses. Consequently, it is plausible that adjunctive treatment with topical antifungal agents could offer a therapeutic advantage by targeting residual fungal disease. The ideal infusion dwell time in cats is currently not known, with a reported range, across both canine and feline patients, of 5 mins to 1 h.^4,6,15,18–20^ A shorter dwell time of 15 mins was chosen with due consideration to the accumulative general anaesthetic time of previous procedures; 15 mins was based on the shorter times reported in canine patients.^15^

Although anterograde endoscopic examination and debridement of the frontal sinuses is described in dogs, this approach is not feasible in cats owing to the narrow aperture of the naso-frontal ostium and the density and vascularity of the ethmoid turbinates.^21^ However, in light of the imaging in this case, evaluation of the frontal sinuses was considered important and sinusotomy was performed to facilitate exploration, debridement and topical administration of clotrimazole solution. Nonetheless, exploration and debridement of the caudal nasal cavity and ethmoid turbinates are practically challenging; it might be hypothesised that the persistence of fungal lesions or secretions in these areas were influential factors in the outcome of this case.

The need for systemic antifungal therapy is debated and, in the absence of a strong evidence base, recommendations are often conflicting and based on expert opinion. For example, while some guidelines on SNA state that systemic antifungals are required in combination with local therapy, others recommend their use only if there is histological evidence of mucosal invasion with fungal hyphae or culture of *A felis.*^4,6,8^ The reported clinical response in two cats with SOA using combined systemic antifungal therapy alone further supports the use of systemic antifungals, particularly when there is concern regarding invasive fungal species.^3^ Itraconazole was used in this case based on UK prescribing regulations and previous reports of its efficacy alongside debridement; however, the optimal antifungal agents and treatment duration are unknown. Likewise, the value of combination therapy compared with treatment with debridement or topical therapy alone, and the influence of the causative fungal species on treatment outcome require further study.

Evaluating the response to treatment is challenging as structural changes are likely to persist and many owners are reluctant to pursue repeat imaging, rhinoscopy and resampling due to the associated morbidity and financial considerations. For this reason, quantitative PCR on nasal swab samples has recently been evaluated for monitoring the treatment outcome in dogs with SNA, with a reported sensitivity of 70% and specificity of 96.2%.^22^ This has not been evaluated in cats and is likely to prove practically challenging in this species. In addition, as seen in this case, PCR may be falsely negative despite a positive culture due to various inhibitory factors unique to the fungus that substantially reduce the amount of fungal DNA detected.^23,24^ Clinical signs are therefore often used as a surrogate to evaluate treatment efficacy; however, the persistence of fungal disease in this case suggests that the resolution of clinical signs cannot be relied upon as a reliable marker of disease cure. Further research is required to better define the outcomes of cats with fungal rhinitis and to determine the optimal means of evaluating treatment response.

## Conclusions

The treatment of feline SNA by endoscopic and surgical debridement, in combination with topical instillation of 1% clotrimazole solution, and systemic itraconazole therapy, resulted in clinical remission of SNA in this case. However, post-mortem investigations confirmed persistent infection, indicating that clinical signs do not necessarily correlate with disease status and may be an unreliable indicator of treatment efficacy. Further studies are warranted to corroborate these findings, establish the optimal treatment regimen and means of evaluating treatment outcome, and determine if disease progression and treatment response differ according to the underlying fungal aetiology.
